# Editorial: Insights in neuropathic pain: 2022

**DOI:** 10.3389/fpain.2023.1232025

**Published:** 2023-06-14

**Authors:** Leyan Shan, Dinesh Selvarajah, Zilong Wang

**Affiliations:** ^1^ Department of Medical Neuroscience, School of Medicine, Southern University of Science and Technology, Shenzhen, China; ^2^ Department of Oncology and Human Metabolism, University of Sheffield, Sheffield, United Kingdom

**Keywords:** neuropathic pain, neuropathy, fatty acids metabolites, diabetic neuropathy, HIV-associated distal neuropathic pain, chemotherapy-induced peripheral neurotoxicity

**Editorial on the Research Topic** Insights in neuropathic pain: 2022

Neuropathic pain refers to a type of chronic that arises from damage or dysfunction in the sensory nervous system (1). It affects approximately 7%–10% of the general population (2). Although extensive research on neuropathic pain has been done, the underlying mechanisms of development and maintenance are still not clear (3, 4). Moreover, neuropathic pain is difficult to treat due to the lack of effective drugs and treatment methods. In this special Research Topic “Highlights in Neuropathic Pain”, we collated six articles that provide new knowledge from respect views about neuropathic pain.

The first review article by Velzen et al. highlights the challenges associated with neuropathic pain, including its prevalence among a large and diverse population of patients, as well as the instability of efficacy due to unacceptable side effects and variations in disease characteristics and patient severity (5). Despite these challenges, there are several opportunities for improvement in the field, including: first, enhancing diagnostic tools: improving or developing new instruments that are more sensitive and accurate at a technical level, such as confocal cornea microscopy (CCM) for efficient diagnosis of small fiber neuropathy. Second, exploring new pharmacological treatments: investigating treatment options based on relevant targets implicated in neuropathic pain, such as monoamine transporters, calcium channels, and cannabinoid receptors. Last, personalizing treatment approaches: tailoring treatment based on detailed patient phenotypes to provide more targeted and effective interventions.

The subsequent review by Meregalli et al. focuses on the involvement of skin innervation in chemotherapy-induced neuropathic pain (6). Neuropathic pain is a common neurological complication of chemotherapy-induced peripheral neurotoxicity (CIPN). The mechanism of CIPN formation is thought to involve axon swelling and mitochondrial accumulation in the early stage, followed by dermal nerve fiber loss and Schwann cell degeneration in the late stage. Various events contribute to the development and maintenance of neuropathic pain in CIPN, including the loss, degeneration, and regeneration of nerve fibers supplying the skin. Chemotherapy-induced mitochondrial damage, reduced cellular bioenergy, increased release of nitric oxide and superoxide, and involvement of other cells like keratinocytes and Langerhans cells are also implicated in neuropathic pain in CIPN. These results suggested a strategy focusing on the complex neuroimmune interactions in the skin for the development of topical analgesics and neuroprotective drugs in CIPN.

The research conducted by Kang et al. explores the relationship between neuropathic pain and radiological patterns of optic neuritis subtypes, specifically first-episode aquaporin-4 (AQP4) antibody-associated ON (AQP4-ON), myelin oligodendrocyte glycoprotein (MOG) antibody-associated ON (MOG-ON), and idiopathic demyelinating optic neuritis (IDON) (7). Magnetic resonance imaging (MRI) scanning and clinical characteristic comparisons were performed to gain insights into the pain and radiological features of different types of optic neuritis. The study indicated that pain is a common symptom in patients with all types of demyelinating ON, suggesting pain treatment should consider as one of the therapeutic objectives in the treatment of ON.

The study by Weeks et al. investigated the mechanism of HIV-associated distal neuropathic pain (DNP) by examining the functional connectivity alterations in the salience network (SN) and default mode network (DMN) using functional neuroimaging: resting state functional magnetic resonance imaging (rs-fMRI) (8). Their results revealed decreased connectivity between the medial prefrontal cortex (MPFC) and posterior cingulate cortex (PCC), representing DMN regions, but stronger connectivity between the anterior cingulate cortex (ACC) and thalamus, indicating increased SN and DMN region connection. These findings contribute to understanding the potential mechanisms involved in the development and maintenance of DNP in the central nervous system.

Yamamoto et al.'s research article evaluated the lipidomic analysis of fatty acids (FAs) metabolites changes over time following peripheral nerve injury (PNI) (9). Their results demonstrate alterations in fatty acid metabolism after nerve injury, especially, increased *ω*-6 FA and *ω*-3 FA metabolites, providing new insights into the pathophysiology and pathologies of neuropathic pain, which may provide potential targets for neuropathic pain treatment.

Lastly, Gandhi et al.'s study offered a novel perspective on neuronal function in diabetic peripheral neuropathy (DPN) (10). Using proton Magnetic Resonance Spectroscopy (1H-MRS), significant differences in cerebral alterations were observed between painful and painless DPN in the thalamus rather than the primary somatosensory (S1) cortex, indicating a strong association between neuropathic pain in DPN and thalamic functions, which might be a potential therapeutic target for new treatments of neuropathic pain in DPN.
